# AMPK/FOXO3a Pathway Increases Activity and/or Expression of ATM, DNA-PKcs, Src, EGFR, PDK1, and SOD2 and Induces Radioresistance under Nutrient Starvation

**DOI:** 10.3390/ijms241612828

**Published:** 2023-08-15

**Authors:** Yusuke Urushihara, Takuma Hashimoto, Yohei Fujishima, Yoshio Hosoi

**Affiliations:** 1 Department of Radiation Biology, School of Medicine, Tohoku University, Sendai 980-8575, Japan; 2 Kobe Research Lab, Oncolys BioPharma Inc., Kobe 650-0047, Japan; 3 Department of Risk Analysis and Biodosimetry, Institute of Radiation Emergency Medicine, Hirosaki University, Hirosaki 036-8562, Japan

**Keywords:** radiation sensitivity, nutrient starvation, AMPK, FOXO3a, ATM, DNA-PKcs, EGFR, PDK1, SOD2, apoptosis

## Abstract

Most solid tumors contain hypoxic and nutrient-deprived microenvironments. The cancer cells in these microenvironments have been reported to exhibit radioresistance. We have previously reported that nutrient starvation increases the expression and/or activity of ATM and DNA-PKcs, which are involved in the repair of DNA double-strand breaks induced by ionizing radiation. In the present study, to elucidate the molecular mechanisms underlying these phenomena, we investigated the roles of AMPK and FOXO3a, which play key roles in the cellular response to nutrient starvation. Nutrient starvation increased clonogenic cell survival after irradiation and increased the activity and/or expression of AMPKα, FOXO3a, ATM, DNA-PKcs, Src, EGFR, PDK1, and SOD2 in MDA-MB-231 cells. Knockdown of AMPKα using siRNA suppressed the activity and/or expression of FOXO3a, ATM, DNA-PKcs, Src, EGFR, PDK1, and SOD2 under nutrient starvation. Knockdown of FOXO3a using siRNA suppressed the activity and/or expression of AMPKα, ATM, DNA-PKcs, FOXO3a, Src, EGFR, PDK1, and SOD2 under nutrient starvation. Nutrient starvation decreased the incidence of apoptosis after 8 Gy irradiation. Knockdown of FOXO3a increased the incidence of apoptosis after irradiation under nutrient starvation. AMPK and FOXO3a appear to be key molecules that induce radioresistance under nutrient starvation and may serve as targets for radiosensitization.

## 1. Introduction

Most solid tumors contain cells in hypoxia, and these hypoxic cells show resistance to ionizing radiation [1]. Hypoxic environments are often associated with nutrient starvation. We have previously reported that nutrient starvation induced radioresistance and that nutrient starvation and hypoxia act synergistically in the induction of radioresistance [2,3]. We have also reported that HIF-1α is not accumulated in cells under hypoxia combined with nutrient starvation [3]. Radioresistance of tumor cells causes poor prognosis after radiotherapy; therefore, it is important to investigate the molecular mechanisms leading to radioresistance under hypoxia and/or nutrient starvation to overcome it [4]. We have reported, as a molecular mechanism for radioresistance under nutrient starvation, increased activity and expression of ataxia-telangiectasia mutation (ATM) and the DNA-dependent protein kinase catalytic subunit (DNA-PKcs), both of which are involved in DNA double-strand break repair [2,3,5]. We have also reported that adenosine monophosphate-activated kinase (AMPK) activated ATM under nutrient starvation [2,3]. Under severe hypoxia, AMPK has been reported to increase the expression of ATM through the transcriptional factor Sp1 and induce radioresistance [6,7]. The molecular mechanisms for the increased expression of ATM and DNA-PKcs under nutrient starvation remain to be elucidated.

AMPK is a cellular energy sensor capable of detecting shifts in the AMP/ATP ratio, activated when cellular energy is low. Cellular energy is low under hypoxia and under nutrient starvation, and AMPK is activated in these conditions. AMPK is a heterotrimeric protein complex composed of α, β, and γ subunits, and the phosphorylation at Thr 172 of AMPKα induces AMPK activation [8]. Recently, several studies reported the involvement of AMPK in DSB repair [9,10]. AMPK has been reported to activate ATM through forkhead box O transcription factor 3a (FOXO3a) activation [11]. AMPK activates FOXO3a by phosphorylation at Thr 179, Ser 399, Ser 413, Ser 555, Ser 588, and Ser 626, and the activated FOXO3a activates ATM via phosphorylation at Ser 1981 [12,13]. On the contrary, it has been reported that ATM activates AMPK via liver kinase B1 (LKB1) in a dependent or independent manner [14,15]. LKB1 has been reported to activate AMPKα by phosphorylation at Thr 172 [15].

FOXO3a, also known as FOXO3, belongs to the family of FOXOs, characterized by a distinct forkhead DNA-binding domain. The FOXO family members consist of FOXO1, FOXO3, FOXO4, and FOXO6 and are the mammalian orthologues of Caenorhabditis elegans (*C. elegans*) DAF-16, which activates genes involved in longevity, lipogenesis, heat shock survival, and oxidative stress responses [16]. DAF-16 is activated under dietary restriction and plays a critical role in the prolonging of the life span of *C. elegans* induced by dietary restrictions. Regarding DNA repair, FOXO3a phosphorylates ATM at Ser 1981 and facilitates ATM-mediated signaling and DNA repair [12].

Although DNA double-strand break repair is the most important determinant of radiosensitivity, other factors also affect radiosensitivity. The epidermal growth factor receptor (EGFR) is a transmembrane tyrosine kinase receptor that constitutes the EGFR family, which consists of EGFR (ErbB-1), HER2 (ErbB-2), Her 3 (ErbB-3), and Her 4 (ErbB-4) [17]. EGFR is overexpressed in a variety of tumor cell lines, and high EGFR expression is associated with poor prognosis and low survival rates. Regarding radiosensitivity, the activation of the EGFR signaling pathway in tumor cells induces radioresistance [17]. Src and phosphoinositide-dependent kinase 1 (PDK1) are involved in EGFR signaling pathways, and their activation is reported to lead to radioresistance of tumor cells [18,19].

Another factor affecting radiosensitivity is superoxide dismutase 2 (SOD2), also known as MnSOD, a mitochondrial protein that forms a homotetramer and binds one manganese ion per unit. SOD2 converts the superoxide anion radical (O_2_^−^) into hydrogen peroxide (H_2_O_2_) and diatomic oxygen (O_2_). Ionizing radiation produces superoxide anion radicals, hydroxyl radicals (·OH), and hydrogen peroxide, which act as mediators of radiation-induced cellular damage. Additionally, SOD2 plays an anti-apoptotic role against ionizing radiation and oxidative stress [14].

In the present study, we investigated the effects of AMPK and FOXO3a on the activity and expression of ATM, DNA-PKcs, EGFR signaling components, and SOD2 under nutrient starvation using MDA-MB-231. The results show the following: (i) nutrient starvation increased the activity and expression of AMPK, FOXO3a, ATM, DNA-PKcs, Src, EGFR, and PDK1 and increased expression of SOD2; (ii) knockdown of AMPKα or FOXO3a suppressed the activity and expression of AMPKα, FOXO3a, ATM, DNA-PKcs, Src, EGFR, and PDK1 and suppressed the expression of SOD2; and (iii) FOXO3a knockdown increased the incidence of radiation-induced apoptosis under nutrient starvation. The results presented in this study suggest that AMPK and FOXO3a appear to be key molecules that induce radioresistance under nutrient starvation.

## 2. Results

### 2.1. Nutrient Starvation-Induced Radioresistance in MDA-MB-231 Cells

**Figure 1:**
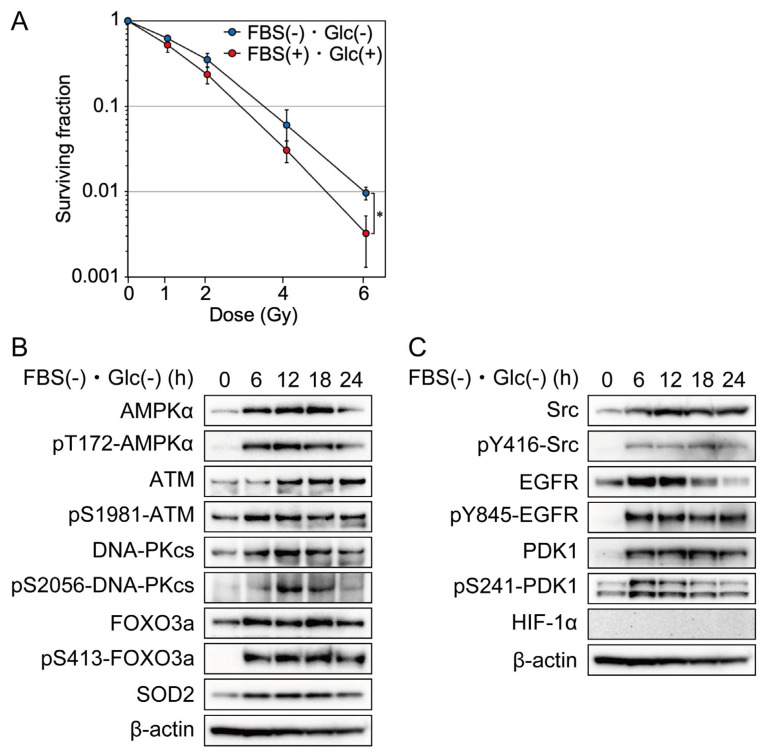
Effects of nutrient starvation on clonogenic cell survival and phosphorylation and/or expression of AMPKα, ATM, DNA-PKcs, FOXO3a, Src, EGFR, PDK1, SOD2, and HIF-1α in MDA-MB-231 cells. (**A**) MDA-MB-231 cells were cultured in glucose-free DMEM without FBS for nutrient starvation (FBS(−) and Glc(−)) or control DMEM with 10% FBS (FBS(+) and Glc(+)) for 12 h before 0–6 Gy irradiation. After irradiation, the cells were cultured under the same condition for 4 h and used for a colony formation assay. The data are presented as the mean ± SD from three independent experiments (*: *p* < 0.05). (**B**,**C**) MDA-MB-231 cells were cultured under nutrient starvation for 0–24 h and processed for Western blot analyses with the antibodies indicated. Western blot results at the 0-h time point showed the expression of AMPKα, ATM, DNA-PKcs, FOXO3a, SOD2, Src, EGFR, PDK1, and HIF-1α when the cells were cultured in the control DMEM containing 1.0 g/L glucose with 10% FBS.

First, we investigated the effects of nutrient starvation on clonogenic cell survival after irradiation with a colony formation assay using MDA-MB-231 cells. Cells were cultured under nutrient starvation or the control conditions for 12 h before irradiation. Four hours after irradiation, the cells were trypsinized into a single-cell suspension and used for the colony formation assay. Cells cultured under nutrient starvation showed a significant increase in clonogenic cell survival after irradiation compared with those under the control condition (Figure 1A).

### 2.2. Effects of Nutrient Starvation on AMPKα, FOXO3a, ATM, DNA-PKcs, SOD2, Src, EGFR, PDK1, and HIF-1α

AMPK is a heterotrimeric protein complex formed by α, β, and γ subunits. The phosphorylation of AMPKα at Thr 172 was examined to evaluate AMPKα activation [20]. Nutrient starvation increased the phosphorylation and expression of AMPKα in MDA-MB-231 cells (Figure 1B). The phosphorylation of FOXO3a at Ser 413 was examined to evaluate FOXO3a activation [21]. Nutrient starvation increased the phosphorylation and expression of FOXO3a in MDA-MB-231 cells (Figure 1B). To investigate the molecular mechanism underlying cellular radioresistance under nutrient starvation, we investigated the effects of nutrient starvation on the activity and expression of ATM and DNA-PKcs, both of which play important roles in the repair of DNA double-strand breaks induced by ionizing radiation. Phosphorylations of ATM at Ser 1981 and DNA-PKcs at Ser2056 were examined to evaluate ATM or DNA-PKcs activation, respectively [22]. As shown in Figure 1B, nutrient starvation increased the phosphorylation and expression of ATM and DNA-PK. Next, we investigated the effects of nutrient starvation on the expression of SOD2. Reactive oxygen species (ROS) mediate ionizing radiation-induced cellular damage, and SOD2 plays an important role in protection against ROS produced by ionizing radiation [23]. As shown in Figure 1B, nutrient starvation increased the expression of SOD2. EGFR signaling has been reported to be involved in DNA double-strand break repair and in mediation of cellular resistance to radiation [24]. In the present study, we investigated the effects of nutrient starvation on the activity and expression of Src, EGFR, and PDK1, which were involved in the EGFR signaling pathway and related to radioresistance [18,19]. Phosphorylations of Src at Tyr 416, EGFR at Tyr 845, and PDK1at Ser241 were examined to evaluate Src, EGFR, or PDK1 activation, respectively [25,26,27]. As shown in Figure 1C, nutrient starvation increased the phosphorylation and expression of Src, EGFR, and PDk1. Hypoxia-inducible factors (HIFs) are transcription factors that respond to hypoxia. Recent studies have revealed that the phosphatidylinositol 3-kinase (PI3K)-Akt pathway mediates non-hypoxic HIF regulation [28]. Because EGFR signaling was activated under nutrient starvation, as shown in Figure 1C, we investigated the effect of nutrient starvation on HIF-1α accumulation using the same antibody that we used to show the accumulation of HIF-1α under hypoxia and lack of accumulation of HIF-1α under hypoxia combined with nutrient starvation using LM217 and HepG2 cells [3]. As shown in Figure 1C, HIf-1α was not accumulated under nutrient starvation.

### 2.3. AMPKα Knockdown Suppressed Activity and/or Expression of ATM, DNA-PKcs, Src, EGFR, PDK1, and SOD2 under Nutrient Starvation

**Figure 2:**
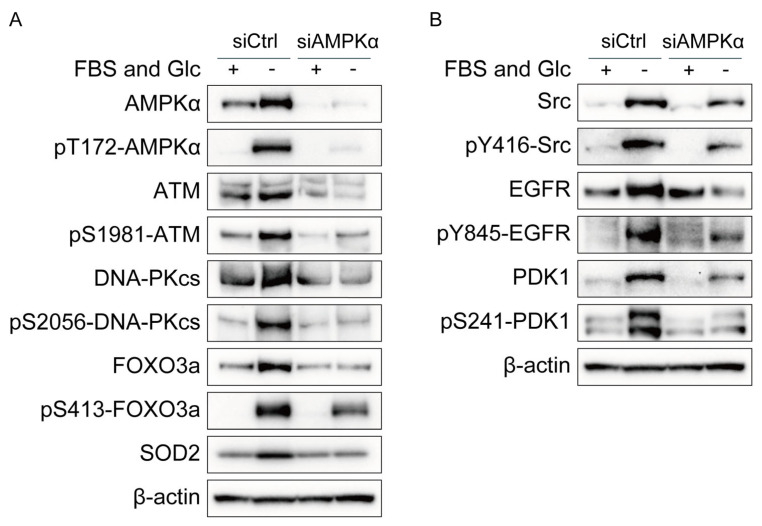
Effects of AMPKα knockdown on phosphorylation and/or expression of ATM, DNA-PKcs, FOXO3a, Src, EGFR, PDK1, and SOD2 in MDA-MB-231 cells. (**A**,**B**) MDA-MB-231 cells treated with siRNA for AMPKα (siAMPKα) or with control siRNA (siCtrl) were cultured in glucose-free DMEM without FBS for nutrient starvation (FBS(−) and Glc(−)) or in control DMEM with 10% FBS (FBS(+) and Glc(+)) for 12 h and processed for Western blot analyses with the antibodies indicated.

Next, we investigated the mechanism underlying the increase in phosphorylation and/or expression of ATM, DNA-PKcs, Src, EGFR, PDK1, and SOD2 under nutrient starvation. Knockdown of AMPKα suppressed the phosphorylation and expression of DNA-PKcs, Src, EGFR, and PDK1 and the expression of SOD2 under nutrient starvation, as shown in Figure 2A,B. The effects of AMPKα knockdown were complicated in detail (Figure 2A,B). Under the AMPKα silencing, the expression of ATM, FOXO3a, and EGFR did not respond to nutrient starvation or was decreased but their phosphorylation responded to nutrient starvation, which may suggest that their phosphorylation response to nutrient starvation was unchanged by the AMPKα silencing. Neither expression nor phosphorylation of DNA-PKcs responded to nutrient starvation under the AMPKα silencing. Both expression and phosphorylation of Src responded to nutrient starvation under the AMPKα silencing. Under the AMPKα silencing, the expression of PDK1 partly responded to nutrient starvation, but its phosphorylation did not respond to nutrient starvation, which suggests that AMPKα increased both expression and phosphorylation of PDK1 under nutrient starvation.

### 2.4. FOXO3a Knockdown Suppressed Activity and/or Expression of ATM, DNA-PKcs, Src, EGFR, PDK1, and SOD2 under Nutrient Starvation

**Figure 3:**
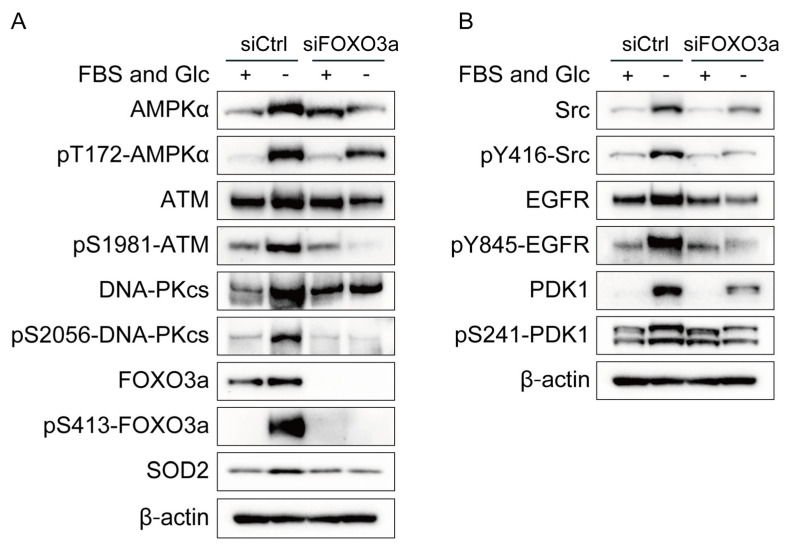
Effects of FOXO3a knockdown on phosphorylation and/or expression of AMPKα, ATM, DNA-PKcs, Src, EGFR, PDK1, and SOD2 in MDA-MB-231 cells. (**A**,**B**) MDA-MB-231 cells treated with siRNA for FOXO3a (siFOXO3a) or with control siRNA (siCtrl) were cultured in glucose-free DMEM without FBS for nutrient starvation (FBS(−) and Glc(−)) or in control DMEM with 10% FBS (FBS(+) and Glc(+)) for 12 h and processed for Western blot analyses with antibodies indicated.

The increased expression of ATM, DNA-PKcs, Src, EGFR, PDK1, and SOD2 under nutrient starvation suggests enhanced transcription of ATM, DNA-PKcs, Src, EGFR, PDK1, and SOD2 under nutrient starvation. FOXO3a is a transcriptional factor activated by nutrient starvation that upregulates the expression of many genes, including ATM [12]. We investigated the effects of FOXO3a knockdown on the phosphorylation and/or expression of ATM, DNA-PKcs, Src, EGFR, PDK1, and SOD2 under nutrient starvation. FOXO3a knockdown suppressed the phosphorylation and expression of ATM, DNA-PKcs, Src, EGFR, and PDK1 and the expression of SOD2 under nutrient starvation (Figure 3A,B). The effects of FOXO3a knockdown were complicated in detail (Figure 3A,B). Under the FOXO3a silencing, the expression of AMPKα was decreased under nutrient starvation, but its phosphorylation responded to nutrient starvation, which may suggest that its phosphorylation response to nutrient starvation was unchanged by the FOXO3a silencing. Under the FOXO3a silencing, the expression of ATM and EGFR was slightly decreased under nutrient starvation, but their phosphorylation was decreased more obviously under nutrient starvation, which suggests that FOXO3a increased both expression and phosphorylation of ATM and EGFR under nutrient starvation. Neither expression nor phosphorylation of DNA-PKcs responded to nutrient starvation under the FOXO3a silencing. Both expression and phosphorylation of Src responded to nutrient starvation under the FOXO3a silencing. Under the FOXO3a silencing, the expression of PDK1 partly responded to nutrient starvation, but its phosphorylation did not respond to nutrient starvation, which suggests that FOXO3a increased both expression and phosphorylation of PDK1 under nutrient starvation.

### 2.5. Mutual Interaction between AMPKα and FOXO3a

AMPK has been reported to modulate the transcriptional activity of FOXO3a [29]. As shown in Figure 2A, AMPKα knockdown suppressed the phosphorylation and expression of FOXO3a. On the contrary, FOXO3a knockdown suppressed the phosphorylation and expression of AMPKα (Figure 3A). These results suggest mutual interaction between AMPKα and FOXO3a.

### 2.6. Effects of FOXO3a Knockdown on Cellular Radiosensitivity

**Figure 4:**
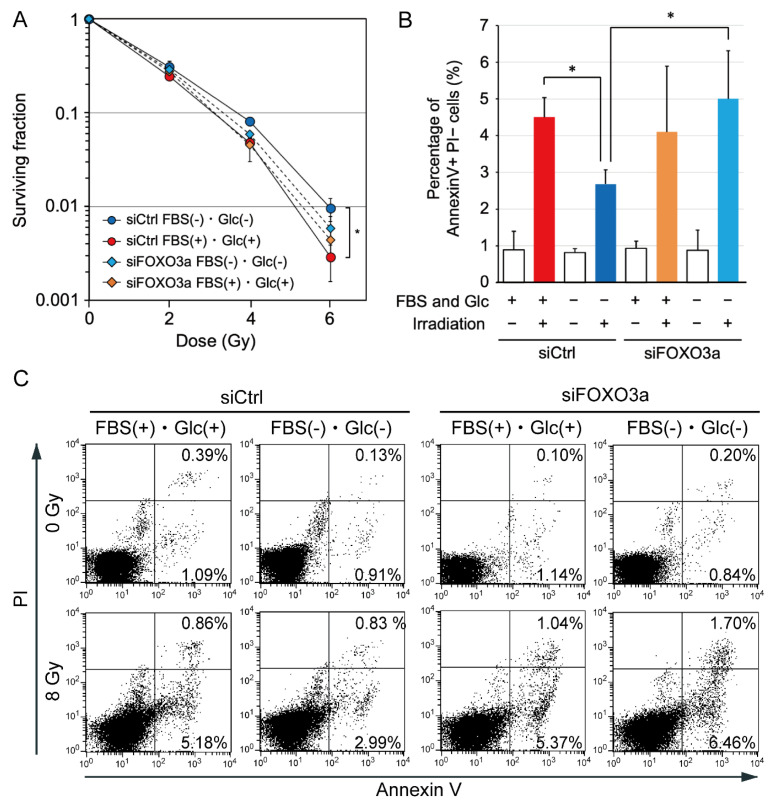
Effects of FOXO3a knockdown on clonogenic cell survival after irradiation and the incidence of radiation-induced apoptosis under nutrient starvation in MDA-MB-231 cells. (**A**) Effects of FOXO3a knockdown on clonogenic cell survival after irradiation under nutrient starvation. MDA-MB-231 cells treated with siRNA for FOXO3a (siFOXO3a) or with control siRNA (siCtrl) were cultured in glucose-free DMEM without FBS for nutrient starvation (FBS(−) and Glc(−)) or in control DMEM with 10% FBS (FBS(+) and Glc(+)) for 12 h before 0–6 Gy irradiation. After irradiation, cells were cultured under the same conditions for 4 h and used for a colony formation assay. The data are presented as the mean ± SD from three independent experiments (*: *p* < 0.05). (**B**) Effects of FOXO3a knockdown on the incidence of radiation-induced apoptosis under nutrient starvation. MDA-MB-231 cells treated with siRNA for FOXO3a or control siRNA were cultured in glucose-free DMEM without FBS for nutrient starvation or control DMEM with 10% FBS for 12 h before 8 Gy irradiation. After irradiation, cells were cultured under the same conditions for 4 h, and then, the medium was replaced with DMEM containing 10% FBS. Twenty hours later, the cells were processed for an annexin V apoptosis assay. The bar graph represents the mean percentage of early apoptotic cells that were positive for annexin V and negative for propidium iodide (PI). The data are presented as the mean ± SD from four independent experiments (*: *p* < 0.05). (**C**) Representative flow cytometry plots using annexin V-FITC/PI staining for apoptosis. A representative result of four experiments is shown. The horizontal axis represents log fluorescence intensity with annexin V staining. The vertical axis represents log fluorescence intensity with PI staining. The lower-left quadrants of the panels show intact viable cells, which were negative for annexin V and PI staining. The lower-right quadrants represent early apoptotic cells, which were positive for annexin V and negative for PI. The upper-right quadrants represent late apoptotic and early necrotic cells, which were positive for annexin V and PI. Each value shown in the panels represents the percentage of cells in the quadrant.

We have previously reported that AMPKα knockdown decreased clonogenic cell survival after irradiation under hypoxia and nutrient starvation [3]. In the present study, we investigated the effects of FOXO3a knockdown on clonogenic cell survival assessed using a colony formation assay and the incidence of apoptosis after 8 Gy irradiation. FOXO3a knockdown tended to decrease clonogenic cell survival after irradiation under nutrient starvation (Figure 4A). The incidence of radiation-induced apoptosis was decreased under nutrient starvation (Figure 4B,C). FOXO3a knockdown significantly increased the incidence of radiation-induced apoptosis under nutrient starvation (Figure 4B,C).

## 3. Discussion

In the present study, we showed that nutrient starvation increased the expression of ATM, DNA-PKcs, Src, EGFR, PDK1, and SOD2 and that knockdown of AMPKα or FOXO3a suppressed the increased expression (Figure 1, Figure 2 and Figure 3). ATM and SOD2 have been reported to be transcriptional targets of FOXOs [16,30,31]. Considering that AMPK activates FOXO3a via phosphorylation at thr179, Ser 399, Ser 413, Ser555, Ser 588, and Ser 626, it is suggested that AMPK increases the expression of ATM, DNA-PKcs, Src, EGFR, PDK1, and SOD2 by the direct activation of FOXO3a under nutrient starvation [12,13,29].

Both hypoxia and nutrient starvation contribute to tumor radioresistance. Oxygen is required for the production of ATP via oxidative phosphorylation. Hypoxia reduces ATP production and increases the AMP/ATP ratio. Similar to hypoxia, nutrient starvation reduces ATP production and increases the AMP/ATP ratio. AMPK is an enzyme that plays a role in cellular energy homeostasis. AMPK is activated when cellular energy states are low and the AMP/ATP ratio is high and is deactivated when cellular energy states are high and the AMP/ATP ratio is low. Therefore, AMPK is activated both under hypoxia and under nutrient starvation. The cellular responses to hypoxia and nutrient starvation are similar in some respects: the expression of AMPK, ATM, DNA-PKcs, and EGFR is increased both under hypoxia and under nutrient starvation (Figure 1B,C) [3,5]. However, the mechanisms underlying the increased expression differ between hypoxia and nutrient starvation. The transcription factor Sp1 increases the expression of ATM, Src, and EGFR under hypoxia, whereas FOXO3a increases the expression of ATM, DNA-PKcs, Src, and EGFR under nutrient starvation (Figure 2 and Figure 3) [6].

Several studies have reported that FOXO3a is involved in the response to DNA damage in a non-transcriptional manner [12,32,33]. The phosphatidylinositol 3-kinase-related kinase (PIKK) family members, including ATM, DNA-PKcs, and ATR, function in signal transduction pathways that activate the DNA damage response [34]. PIKK family members contain conserved C-terminal FAK focal adhesion targeting (FAT) and FATC domains [34]. Tsai et al. reported that the C-terminal domain of FOXO3a binds to ATM’s FAT domain and promotes ATM phosphorylation at Ser 1981 [12]. Adamowicz et al. showed that FOXO3a directly binds to the FATC domain of ATM and to a lysine acetyl-transferase 5 (KAT5) (also known as a Tip60) and that the formation of the ATM-FOXO3a-KAT5/Tip60 complex is necessary for the activation of the DNA damage response [33]. It has been reported that KAT5/Tip60 also interacts with the FATC domain of DNA-PKcs and that the DNA-PKcs-KAT5/Tip60 complex enhances DNA-PKcs activation and the DNA damage response [34,35]. These reports suggest that FOXO3a activates ATM and DNA-PKcs in a non-transcriptional manner under nutrient starvation.

In response to DNA double-strand breaks, ATM and DNA-PKcs are rapidly activated through phosphorylation at serine 1981 and serine 2056, respectively. Then, ATM and DNA-PKcs activate many kinases involved in DNA damage responses [36,37]. ATM initiates DSB repair, and DNA-PKcs phosphorylates many non-homologous end-joining (NHEJ) factors, such as Ku70/80, XRCC4, and DNA ligase IV [38,39]. The expression levels of ATM and DNA-PKcs have been reported to correlate with radiosensitivity [40,41]. These facts suggest that the increased activity and expression of ATM and DNA-PKcs under nutrient starvation observed in the present study contribute to the radioresistance under nutrient starvation.

While the AMPK/FOXO3a pathway is involved in DNA double-strand break repair and radiosensitivity through the increased activity and expression of ATM and DNA-PKcs under nutrient starvation, AMPK has other functions related to DNA double-strand break repair. AMPK is reported to directly phosphorylate 53BP1 at Ser1317 and promote 53BP1 recruitment in the process of NHEJ, which facilitates the end joining of distal DNA ends [10].

Ionizing radiation generates ROS, which show high reactivity with various cellular molecules, including DNA, and cause cytotoxicity, mutagenicity, and apoptosis [42,43]. SOD2 scavenges ROS generated within the mitochondria and inhibits radiation-induced apoptosis through the stabilization of the mitochondrial membrane [23,44]. In the present study, the expression of SOD2 was increased, and the incidence of radiation-induced apoptosis was decreased under nutrient starvation (Figure 1B and Figure 4B). FOXO3a knockdown decreased the expression of SOD2 and reduced the incidence of radiation-induced apoptosis (Figure 4B). These results suggest that increased expression of SOD2 decreases the incidence of radiation-induced apoptosis under nutrient starvation.

**Figure 5:**
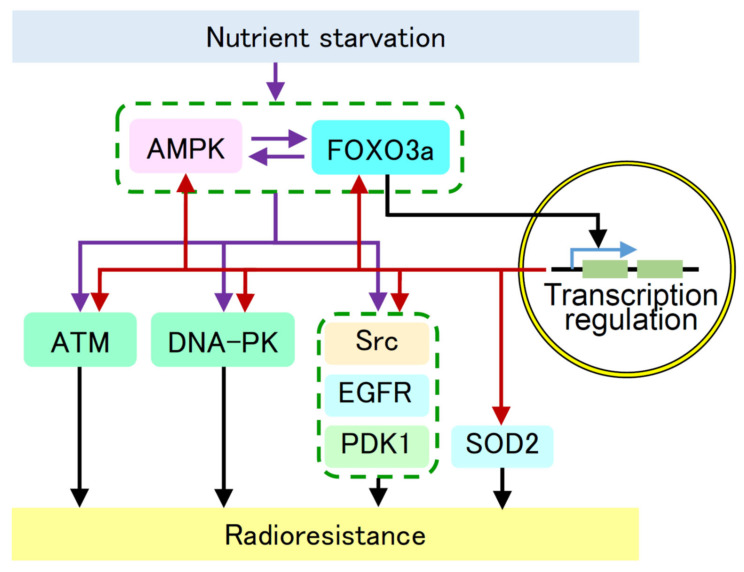
Suggested molecular pathway for the increased activity and/or expression of AMPK, FOXO3a, ATM, DNA-PKcs, Src, EGFR, PDK1, and SOD2 under nutrient starvation. Our results suggest that nutrient starvation activates the AMPK/FOXO3a pathway and induces radioresistance via increased activity and/or expression of ATM, DNA-PKcs, Src, EGFR, PDK1, and SOD2. AMPK and FOXO3a appear to be key molecules that induce radioresistance under nutrient starvation. Purple arrows indicate activation pathways presented in this manuscript. Red arrows indicate transcriptional activation presented in this manuscript. Black arrows indicate previously reported pathways. Blue arrow indicates transcription.

In summary, our findings suggest that the AMPK/FOXO3a pathway increases the activity and/or expression of ATM, DNA-PKcs, Src, EGFR, PDK1, and SOD2 and induces radioresistance under nutrient starvation (Figure 5). AMPK and FOXO3a appear to be key molecules that induce radioresistance under nutrient starvation and may serve as targets for radiosensitization.

## 4. Materials and Methods

### 4.1. Cell Line and Nutrient Starvation Culture

Human MDA-MB-231 breast cancer cells (American Type Culture Collection, Manassas, VA, USA) were cultured in DMEM (#08456-36, Nacalai Tesque, Inc., Kyoto, Japan) containing 10% fetal bovine serum (FBS) (#171012, Nichirei Biosciences Inc., Tokyo, Japan) and penicillin–streptomycin (#09367-34, Nacalai Tesque, Inc.). For nutrient starvation, the cells were cultured in glucose-free DMEM (#042-32255, Wako Pure Chemical Corp., Osaka, Japan) without FBS. For the control condition, the cells were cultured in DMEM with 1.0 g/L glucose and 10% FBS. In this manuscript, “nutrient starvation” refers to culturing in a glucose-free medium without FBS.

### 4.2. Irradiation

X-ray irradiation was performed using an X-ray generator, Model M-150WE (SOFTEX Co., Ltd., Ebina, Japan), at 130 kV-8 mA with a 0.5 mm aluminum filter at a dose rate of 0.56 Gy/min.

### 4.3. Colony Formation Assay

Clonogenic cell survival was assessed using a colony formation assay. Cells were cultured under nutrient starvation or the control condition for 12 h before irradiation. Four hours after irradiation, cells were trypsinized to produce a single-cell suspension, seeded into 60 mm dishes at various cell densities, and cultured with the control medium. After 12 days of culture, the colonies were stained with crystal violet, and the number of colonies containing more than 50 cells was counted.

### 4.4. Annexin V Apoptosis Assay

Cells were cultured under nutrient starvation or the control condition for 12 h before 8 Gy irradiation. Four hours after irradiation, the medium was replaced with the control medium. Twenty hours later, cells were collected with trypsin and washed twice with phosphate-buffered saline. Apoptotic cells were stained using the annexin V-FITC Apoptosis Detection Kit (#15342-54, Nacalai Tesque, Inc.) following the manufacturer’s instructions. The cells were analyzed using the BD FACS Canto II system (BD Biosciences, Franklin Lakes, NJ, USA). Over 10,000 cells were analyzed for each sample.

### 4.5. Western Blot Analysis

Western blot analysis was carried out as previously reported [7]. The following antibodies were used as primary antibodies: AMPKα antibody (#2603, Cell Signaling Technology, Inc. (CST), Beverly, MA, USA); phospho-AMPKα (Thr172) antibody (#2535, CST); ATM antibody (#NB100-104, Novus Biologicals, LLC, Englewood, CO, USA); phospho-ATM (Ser1981) antibody (#5883, CST); DNA-PKcs antibody (#sc-9051, Santa Cruz Biotechnology, Inc., Dallas, TX, USA); phospho-DNA-PKcs (Ser2056) antibody (#ab18192, Abcam, PLC, Cambridge, UK); EGFR antibody (#2232, CST); phospho-EGFR (Tyr845) antibody (#2231, CST); FOXO3a antibody (#12829, CST); phospho-FOXO3a (Ser413) antibody (#8174, CST); hypoxia-inducible factor-1 alpha (HIF-1α) antibody (#A300-286A, Bethyl Laboratories, Inc., Montgomery, TX, USA); PDK1 antibody (#3062, CST); phospho-PDK (Ser241) antibody (#3438, CST); SOD2 antibody (#13141, CST); Src antibody (#2108, CST); and phospho-Src (#2102, CST) antibody. The HRP-conjugated swine anti-rabbit IgG antibody (#P0399, Dako, Ltd., Glostrup, Denmark) was used as a secondary antibody. The monoclonal anti-β-actin-peroxidase antibody (#A3854, Millipore Sigma, Co., St. Louis, MO, USA) was used as a loading control. Detection was performed using the ECL prime reagents (#RPN2232, Cytiva, Co., Tokyo, Japan) or Chemi-Lumi One Ultra (#11644, Nacalai Tesque, Inc.) with a ChemiDoc XRS (Bio-Rad Laboratories, Inc., Hercules, CA, USA).

### 4.6. Small Interfering RNA (siRNA)

RNA silencing was achieved with a siRNA specific for AMPKα1/2 (#sc-45312, Santa Cruz Biotechnology, Inc.) or FOXO3a (#J-003007-09, GE Healthcare Dharmacon, Inc., Lafayette, CO, USA) at a final concentration of 10 nM. The siRNAs were delivered to the cells using RNAiMAX reagent (Invitrogen, Co., Carlsbad, CA, USA) following the manufacturer’s instructions. A non-targeting siRNA (#D-001810, GE Healthcare Dharmacon, Inc.) was used as a control for non-sequence-specific effects in each transfection. Forty-eight hours after adding siRNA, the medium was replaced with the nutrient-starved or control medium.

### 4.7. Statistical Analysis

All data are expressed as means and standard deviations from at least three independent experiments. The statistical significance of the difference in survival fraction and the incidence of apoptotic cells between the two groups was analyzed with Welch’s *t*-test. Any *p*-values less than 0.05 were considered statistically significant.

## Data Availability

The authors confirm that the data supporting the findings of this study are available within the article.
